# The Significance of Autoantibodies in Juvenile Dermatomyositis

**DOI:** 10.1155/2021/5513544

**Published:** 2021-11-19

**Authors:** Dominika Kwiatkowska, Adam Reich

**Affiliations:** Department of Dermatology, Institute of Medical Sciences, Medical College of Rzeszow University, Rzeszów, Poland

## Abstract

Juvenile dermatomyositis is a chronic and rare autoimmune disorder classified into the spectrum of idiopathic inflammatory myopathies. Although this entity is mainly characterized by the presence of pathognomonic cutaneous lesions and proximal muscle weakness, the clinical manifestation can be highly heterogeneous; thus, diagnosis might be challenging. Current treatment recommendations for juvenile dermatomyositis, based mainly upon case series, include the use of corticosteroids, immunomodulatory, and immunosuppressive agents. Recently, several specific autoantibodies have been shown to be associated with distinct clinical phenotypes of classic dermatomyositis. There is a need to further evaluate their relevance in the formation of various clinical features. Furthermore, while providing more personalized treatment strategies, one should consider diversity of autoantibody-related subgroups of juvenile dermatomyositis.

## 1. Introduction

Juvenile dermatomyositis (JDM) is a chronic, systemic, autoimmune disease belonging to the so called connective tissue disorders. It is the most common inflammatory myopathy in children, accounting for approximately 85% of cases. However, it is still a very rare disease [1]. Girls are more often affected than boys, with a ratio of 2.3 : 1, respectively [2]. The mean age of onset is around 7 years. JDM is characterized by the presence of pathognomonic cutaneous lesions and proximal muscle weakness. Typical dermatological features include heliotrope rash and Gottron's papules over the extensor surfaces. However, JDM can be highly heterogeneous, and many other symptoms may be observed during the course of the disease, sometimes making the diagnosis difficult. In adults, elevated serum levels of muscle enzymes, including creatine kinase (CK), transaminases, lactate dehydrogenase (LDH), and aldolase, play an important role in the diagnosis and severity assessment of DM. Nonetheless, those enzymes proved to be weakly correlated with disease severity in JDM [3–5]. The clinical outcome of JDM may vary depending on several comorbidities, such as interstitial lung disease or calcification. In adults, there is a well-established relationship between DM and malignancies. However, the paraneoplastic phenomenon has very rarely been noted in pediatric patients, and the current data are limited to a few cases in the literature [6, 7].

Recently, specific circulating autoantibodies have been shown to be associated with peculiar clinical phenotypes of classic dermatomyositis (DM). However, the prognostic value of these autoantibodies remains to be identified in children with JDM. Currently, recommendations for the treatment of juvenile myositis are based mainly on expert opinions, as data from randomized, controlled trials are very limited [8]. Various clinical phenotypes of JDM seem to present variable responses to treatment; thus, a better understanding of the exact role of specific autoantibodies in the formation of distinct clinical features could be of help creating a more personalized and efficacious treatment strategy.

### 1.1. Clinical Presentation of JDM Correlates with Specific Autoantibodies

Autoantibodies present in DM have been divided into two major groups, myositis-specific autoantibodies (MSAs) and myositis-associated autoantibodies (MAAs). However, the frequency of autoantibodies in JDM is lower than that in adults; it is estimated that these antibodies can be detected in about 60% of JDM cases [9]. Several autoantibodies have been proven to be related to specific clinical presentation and possibly may also be a useful prognostic tool of JDM.

## 2. Materials and Methods

**Figure 1:**
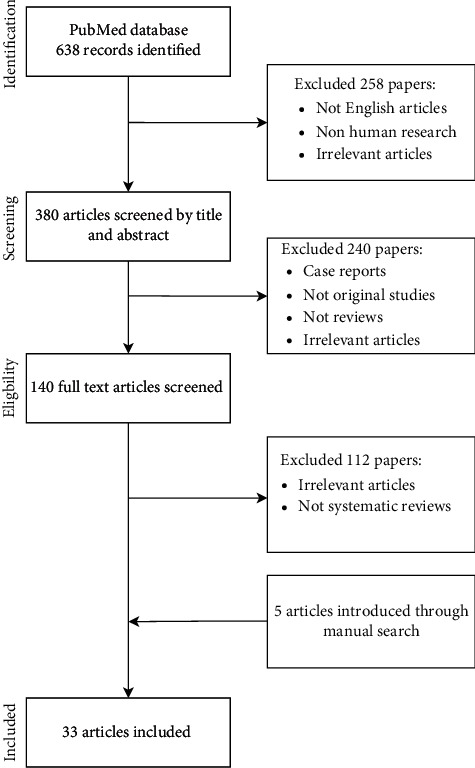
Process of searching the PubMed database.

For the purposes of this article, a detailed search of the PubMed database was carried out, using the following search terms: juvenile dermatomyositis, myositis specific autoanti-bodies, anti-TIF1-*γ* autoantibodies (anti-p155), anti-Mi-2 autoantibodies, anti-MDA5 autoantibodies (anti-CADM-140), anti-NXP2 autoantibodies (anti-MJ antibodies), anti-SAE autoantibodies, anti-SRP autoantibodies, anti-HMGCR autoantibodies, anti-ARS autoantibodies, myositis associated autoantibodies, Anti-PmScl autoantibodies, and anti-U1-ribonucleoprotein autoantibodies (Figure 1). Articles were selected based on their relevance regarding the topic of this review. Several other articles were also retrieved by manual search of references of already included papers. All studies published in languages other than English were excluded. This article is based on previously conducted studies and does not contain any studies with human participants or animals performed by any of the authors.

## 3. Myositis-Specific Autoantibodies

### 3.1. Anti-TIF1-*γ* Phenotype

Autoantibodies against transcriptional intermediary factor 1-gamma (TIF1-*γ*) are of clinical importance in both JDM and adult DM patients (Table 1). These antibodies were originally named as anti-p155 or anti-p155/140 and were identifiable in around 32% of JDM patients. Typically, cutaneous involvement is more prominent in TIF1-*γ*-positive patients and encompasses extensive erythematous lesions that may be presented within the face, neck (V-sign), shoulders (shawl sign), extensor surfaces of the extremities (holster's sign), and dorsal part of hands. Children are at a lower risk of developing V-sign than adults. However, the presence of this rash is higher than in other autoantibody subgroups. Pediatric patients with TIF1-*γ* autoantibodies may present periungual capillary changes. Severe cutaneous manifestations, such as a lipodystrophy and skin ulceration, are more common [10]. This group is also characterized by pronounced photosensitivity and predominantly chronic disease course. The risk of cancer in adult patients with TIF1-*γ*-positive DM has been reported frequently throughout the literature [11]; however, such relationship has not been observed in pediatric patients [12].

### 3.2. Anti-Mi-2 Phenotype

Anti-Mi-2 autoantibodies are directed against Mi-2 protein which is a component of the nucleosome remodeling deacetylase complex (NuRD) [13]. These antibodies have been demonstrated in about 4-10% of JDM patients. According to Rider et al., classic dermatomyositis features such as Gottron's papules, heliotrope rash, shawl sign, and V-sign as well as cuticular overgrowth and carpal tunnel syndrome were more often observed in adults with anti-Mi-2 autoantibodies compared to juvenile cases [14], whereas symptoms as myalgia and arthritis had similar prevalence in both adults and children with anti-Mi-2 autoantibodies. In the pediatric cohort, anti-Mi-2 autoantibodies were associated with greater muscle weakness and dysphagia. The risk of developing interstitial lung disease (ILD) appears to be lower compared to other dermatomyositis phenotypes [15]. This group of patients has a relatively good prognostic profile, due to a good response to conventional medications (Table 1).

### 3.3. Anti-MDA5 Phenotype

Autoantibodies against melanoma differentiation-associated protein 5 (MDA5) were previously reported as anti-CADM-140 antibodies that interacted with a 140 kDa cytoplasmic protein [16]. There are some discrepancies between studies evaluating the prevalence of anti-MDA5 autoantibodies. In the Japanese JDM cohort, anti-MDA5 autoantibodies were found in 23.8-33% of cases [17, 18], whereas in the JDM cohort from the United Kingdom, these autoantibodies were identified in only 7% of patients [19]. These differences could be explained by the diversity in ethnicity or environmental background. The dermatological features of anti-MDA5-positive JDM include erythematous papules and macules on the palmar surfaces of the metacarpal and interphalangeal joints which, contrary to Gottron's papules, were often painful. Other findings are cutaneous ulcerations located on the lateral nailfolds as well as over the elbows and knees [19]. There is also some evidence for the occurrence of mechanic's hands, fever, and arthritis [20–22]. Arthritis is often symmetrical, affecting the small joints of the hands, and is associated with morning stiffness [19]. Anti-MDA5 autoantibodies have been described to be associated with many cases of amyopathic dermatomyositis presenting rapidly progressive ILD. It is worth noting, that in one case report of children with anti-MDA5 autoantibodies, infection of COVID-19 turned out to be fatal due to serious pulmonary complications [23]. This subset of patients may require more intensive and complex immunosuppressive therapy, as JDM patients with anti-MDA5 autoantibody-associated ILD are often refractory to conventional treatment strategies (Table 1).

### 3.4. Anti-NXP2 Phenotype

The antinuclear matrix protein 2 (NXP2) antibodies, formerly known as anti-MJ antibodies, are correlated with severe muscle weakness, joint contractures, intestinal vasculitis, and polyarthritis (Table 1). A recently conducted study exploring the clinicopathological subgroups of JDM showed that anti-NXP2 antibodies are linked to higher clinical severity, due to increased risk of gastrointestinal bleeding, ulcers, and dysphagia. It was observed that low body mass index (BMI) and positive ANA were associated with gastrointestinal involvement and mortality in this group of patients [24]. In research performed by Wang et al., a lowered CD4/CD8 ratio (*p* = 0.0255) and high ferritin level (*p* = 0.0361) appeared to correlate with more refractory cases [3]. Furthermore, serum ferritin was shown to be valuable as a predictor of the occurrence of concomitant ILD [25]. To date, many studies found that patients with anti-NXP-2 autoantibodies are at higher risk of calcinosis; the severity of which is considerably worse in the young children population [26, 27]. Calcinosis is a complication of dermatomyositis more often observed in pediatric patients, and it manifests as deposition of insoluble calcium salts that may be intracutaneous, subcutaneous, fascial, and intramuscular. Sometimes calcinosis can even involve visceral organs. The presence of anti-NXP2 autoantibodies has been shown to increase the risk of calcinosis. In a cohort of 285 pediatric patients, 43% of anti-NXP2-positive patients developed calcinosis compared to 30% of anti-NXP2-negative patients regardless of age (*p* = 0.025) [27].

### 3.5. Anti-SAE Phenotype

Autoantibodies to small ubiquitin-like modifier activating enzyme (anti-SAE) were reported in JDM patients with a very low prevalence, of less than 1% of cases (Table 1). In a study performed on the large UK cohort, only three patients out of 380 had anti-SAE autoantibodies, which presence was linked to clinically amyopathic dermatomyositis. It is characterized by pathognomonic cutaneous symptoms of dermatomyositis without muscle involvement. This disease is infrequently recognized within a pediatric group [28, 29]. Two patients presented with persistent JDM rash without muscle involvement. Interestingly, a weakening and an increase in muscle enzymes were later observed. Myositis was diagnosed by clinical examination, raised muscle enzymes, and muscle biopsy findings. Contrarily, the third patient presented with a history of myalgia and weakness with no rash. This patient subsequently developed characteristic dermatomyositis skin manifestations two years later. Thus, individuals who initially have muscle symptoms may later develop hallmark cutaneous symptoms despite achieving remission of muscle disease.

### 3.6. Anti-SRP and anti-HMGCR Phenotype

Autoantibodies directed against signal recognition peptide (SRP) and HMG-CoA reductase (3-hydroxy-3-methyl-glutaryl-coenzyme A reductase (HMGCR)) are related to immune-mediated necrotizing myositis (INM) [30]. These associations led to the distinction of three INM subclasses: anti-SRP positive INM, anti-HMGCR positive INM, and seronegative INM [31].

Anti-SRP-positive INM represents 1.6% of patients with JDM [14]. The clinical presentation encompasses severe muscle weakness, with minimal or no typical JDM rash. The highly elevated creatinine kinase levels found in these cases may indicate disruption of the myofiber membrane due to a severe immune-mediated necrotizing myopathy. Additionally, Raynaud phenomenon, dysphonia, and dyspnea on exertion were reported. There is also a risk of cardiac abnormalities. However, pediatric patients showed a greater ability to regenerate muscles, including the cardiac muscle, thus achieving better results during the follow-up period [8].

To date, the prevalence of anti-HMGCR autoantibodies in patients with JDM is estimated to be around 1% [32]. In the study conducted by Takayuki et al., all pediatric patients with anti-HMGCR autoantibodies presented distal weakness in the wrist and ankle flexors and extensors, falling episodes, muscle atrophy, and fatigue [33]. Moreover, each patient developed arthralgias and joint contractures. Markedly elevated creatinine kinase level was also reported. It is worth mentioning that children with an anti-HMGCR-positive profile exhibited a strong association with HLA DRB1∗07 : 01. In adults, the HLA allele DRB1∗11 : 01 is involved in the pathogenesis of anti-HMGCR DM, which may suggest discrepancies in the epitope reactivity between children and adults [34]. The presence of anti-HMGCR in adults has been shown to be associated with the use of statins [35]. However, this correlation did not turn out to be significant in children.

### 3.7. Anti-ARS Phenotypes

The group of anti-aminoacyl tRNA synthetase (anti-ARS) autoantibodies consists of anti-Jo-1, anti-PL-7, anti-PL-12, anti-EJ, anti-OJ, anti-KS, anti-ZO, and anti-YRS/HA autoantibodies. The presence of the above antibodies is linked to unique clinical features that provided for the distinction of the so-called antisynthetase syndrome. In pediatric populations, this syndrome is rare and only found in approximately 5% of JDM cases [36]. The characteristic features include myositis, ILD, fever, Raynaud's phenomenon, arthritis, and mechanic's hands. There is also a risk for the development of lipoatrophy. Among anti-ARS autoantibodies, anti-Jo-1 is most commonly reported. Moreover, these patients may have a better prognosis, due to lower risk of interstitial lung disease [37].

## 4. Myositis-Associated Autoantibodies (MAAs)

Myositis-associated antibodies (MAAs) are common in children with additional symptoms of various autoimmune connective tissue diseases. JDM can overlap with systemic sclerosis, inflammatory arthritis, or systemic lupus erythematosus. The best-known MAAs in JDM patients are antipolymyositis-scleroderma (PM-Scl) and anti-U1-ribonucleoprotein (RNP) autoantibodies. Anti-PmScl autoantibodies were reported in patients showing clinical features of both myositis and systemic sclerosis (SSc) [38]. Overlap SSc/myositis has a favorable prognosis because of the mild myositis and good response to treatment. Nonetheless, these autoantibodies were found to be linked to the development of calcinosis. Pediatric patients may as well present with intensive skin rashes. Anti-U1-snRNP autoantibodies occurred less likely in JDM and were predominantly identified in patients with lupus and mixed connective tissue disease [39].

## 5. Conclusions

Although JDM is an infrequent autoimmune disease, it represents the most common type of idiopathic inflammatory myopathy during childhood. There is an increasing evidence for the utility of various autoantibodies as prognostic biomarkers in JDM. While providing novel treatment strategies, one should consider diversity between clinical presentations of JDM autoantibody-related subgroups.

## Figures and Tables

**Table 1:** Myositis-specific autoantibodies in juvenile dermatomyositis.

Autoantibody	Autoantigen	Prevalence	Response to treatment	Significance
Anti-TIF1-*γ* (anti-p155)	Transcription intermediary factor 1-*γ*	32%	This subset of patients more often receive treatment with a biologic drugs and/or intravenous cyclophosphamide	Severe cutaneous disease: lipodystrophy, skin ulceration and edema
Ani-Mi-2	Nucleosome remodeling deacetyalse complex (NuRD)	4-10%	Respond well to conventional therapy	Greater muscle weakness, dysphagia, and edema
Anti-MDA5 (anti-CADM-140)	Melanoma differentiation-associated protein 5	7-33%	Frequently requires intensive immunosuppressive therapy combining several immunosuppressants	Rapidly progressive ILD, higher IL-18, IL-6, and ferritin levels
Anti-NXP2	Nuclear matrix protein 2b	15-23%	Required more aggressive treatment with a lower remission rate during the follow-up period	Severe muscle weakness, joint contractures, intestinal vasculitis, polyarthritis, calcinosis
Anti-SAE	Small ubiquitin-like modifier activating enzyme	1%	Respond well to conventional therapy	Amyopathic dermatomyositis
Anti-SRP	Signal recognition peptide (SRP)	1.6%	Poorly responsive to standard treatment, however, with satisfying response to aggressive treatment with a combination of rituximab, cyclophosphamide and IVIGs, followed by maintenance methotrexate and intensive daily physical therapy	Immune-mediated necrotizing myositis, severe muscle disease, cardiac involvement
Anti-HMGCR	HMG-CoA reductase (3-hydroxy-3-methyl-glutaryl-coenzyme A reductase (HMGCR)	1%	Poorly responsive to standard treatment, only partial responses to multiple immunosuppressive medications	Immune-mediated necrotizing myositis, worse disease course
Anti-ARS: (i) Anti-Jo-1 (ii) Anti-PL-7 (iii) Anti-PL-12 (iv) Anti-EJ (v) Anti-OJ (vi) Anti-KS (vii) Anti-Zo (viii) Anti-YRS/Ha	Aminoacyl-tRNA synthetases (ARS): (i) Histidyl-tRNA synthetase (ii) Treonyl-tRNA synthetase (iii) Alanyl-tRNA synthetase (iv) Glycyl-tRNA synthetase (v) Isoleucyl-tRNA synthetase (vi) Asparaginyl-tRNA synthetase (vii) Phenylalanine-tRNA synthetase (viii) Tyrosyl-tRNA synthetase	<5%	Glucocorticoids are the empirical first-line therapy; however, additional immunosuppressive agents are often necessary	Anti-synthetase syndrome; myositis, ILD, fever, Raynaud's phenomenon, arthritis, and mechanic's hands.
